# A survey of *Pireneitega* from Tajikistan (Agelenidae, Coelotinae)

**DOI:** 10.3897/zookeys.635.10487

**Published:** 2016-11-23

**Authors:** Xiaoqing Zhang, Yuri M. Marusik

**Affiliations:** 1 College of Life Science, Shenyang Normal University, Shenyang, Liaoning 110034, China; 2 Institute for Biological Problems of the North RAS, Portovaya Str. 18, Magadan 685000, Russia; 3 Department of Zoology & Entomology, University of the Free State, Bloemfontein 9300, South Africa; 4 Zoological Museum, University of Turku, FI-20014 Turku, Finland

**Keywords:** Aranei, central Asia, description, new species, *Paracoelotes*, redescription, spider, taxonomy

## Abstract

Five new species of *Pireneitega* species from Tajikistan are described: *Pireneitega zonsteini* **sp. n.** (♂♀), *Pireneitega muratovi* **sp. n.** (♀), *Pireneitega tyurai* **sp. n.** (♀), *Pireneitega ramitensis* **sp. n.** (♀) and *Pireneitega kovblyuki* **sp. n.** (♂). *Pireneitega major* (Kroneberg, 1875) is redescribed for the first time based on the lectotype designated here. DNA barcodes for the five new species are documented for future use and as proof of molecular differences between these species.

## Introduction

Coelotinae is the largest subfamily of Agelenidae with more than 650 species distributed in the Holarctic and southeast Asia (World Spider Catalog 2016). *Pireneitega* Kishida, 1955 with 30 species distributed across the Palaearctic (World Spider Catalog 2016, Zhang et al. 2016) is one of the most species-rich genera of the subfamily. It is relatively well studied in comparison to other species-rich (and non-monophyletic) genera *Coelotes* Blackwall, 1841 and *Draconarius* Ovtchinnikov, 1999. The species of *Pireneitega* found in Caucasus and Xinjiang were recently revised (Kovblyuk et al. 2013; Zhang et al. 2016) but the genus remains poorly studied in Central Asia. Of three species known from central Asia (Mikhailov 2013: *Pireneitega birulai* (Ermolajev, 1927) (currently considered a junior synonym of *Pireneitega luctuosa* (L. Koch, 1878)), *Pireneitega fedotovi* (Charitonov, 1946) and *Pireneitega major* (Kroneberg, 1875)), *Pireneitega fedotovi* is known only from the original description and *Pireneitega major* only from two very short descriptions supplied with sketchy figures. A short trip by the junior author to Tajikistan revealed five new morphospecies of *Pireneitega*, each separated by distinct genetic gaps. The goal of this paper is to provide descriptions of the new species (including records of their molecular markers) and a redescription of *Pireneitega major* whose type locality lies in northern Tajikistan.

## Material and methods

Specimens were examined and measured with a Leica M205C stereomicroscope. Images were captured with an Olympus C7070 wide zoom digital camera (7.1 megapixels) mounted on an Olympus SZX12 dissecting microscope. Epigynes and male palps were examined after dissection. Epigynes were cleared by boiling it in 10% KOH solution before taking photos of the dorsal view. All measurements are given in millimeters. *Pireneitega major* was photographed and drawn using an MBS-9 stereomicroscope with Pro-MicroScancamera. Leg measurements are given as: total length (femur, patella + tibia, metatarsus, tarsus).

Terminology used for copulatory organ characters in the text and figure legends follows Wang (2002) with some modifications.

Abbreviations used in the text and figure legends are:

A
 epigynal atrium 


ALE
 anterior lateral eye 


AME
 anterior median eye 


AME-ALE
 distance between AME and ALE 


AME-AME
 distance between AME and AME 


ALE-PLE
 distance between ALE and PLE 


CD
 copulatory ducts 


CF
 cymbial furrow 


CO
 conductor 


d
 dorsal 


E
 embolus 


EB
 embolic base 


ET
 epigynal teeth 


FD
 fertilization ducts 


Fe
 femur 


H
 epigynal hood 


MA
 median apophysis 


Mt
 metatarsus 


p
 prolateral 


PA
 patellar apophysis 


Pa
 patella 


PLE
 posterior lateral eye 


PME
 posterior median eye 


PME-PLE
 distance between PME and PLE 


PME-PME
 distance between PME and PME 


R
 receptacle 


r
 retrolateral 


RTA
 retrolateral tibial apophysis 


ST
 subtegulum 


T
 tegulum 


Ta
 tarsus 


Ti
 tibia 


v
 ventral 


VTA
 ventral tibial apophysis 


DNA barcodes were obtained for future use: a partial fragment of the mitochondrial gene cytochrome oxidase subunit I (COI) was amplified and sequenced for five new species using Primers LCO1490-oono (5’-CWACAAAYCATARRGATATTGG-3’) (Folmer et al. 1994; Miller et al. 2010) and HCO2198-zz (5’-TAAACTTCCAGGTGACCAAAAAATCA-3’) (Folmer et al. 1994; Zhao and Li 2016). For additional information on extraction, amplification, and sequencing procedures, see Zhao et al. (2013). All sequences were blasted in GenBank; accession numbers are provided in Table 1.

**Table 1.** Voucher specimen information.

Species	GenBank accession number	Sequence length	Collection localities
*Pireneitega zonsteini* sp. n.	KY024475	642bp	Env. of Dushanbe, Hissar Mt. Ridge 48^th^ km of Varzob Hwy
*Pireneitega muratovi* sp. n.	KY024477	642bp	Env. of Dushanbe Hissar, Mt. Ridge 20^th^ km of Varzob Hwy Gusgarf Vill.
*Pireneitega tyurai* sp. n.	KY024478	642bp	Khatlon Area Khovaling Distr., Obimazar River
*Pireneitega ramitensis* sp. n.	KY024476	642bp	Khatlon Area Hissar Mt. Range Ramit Reserve
*Pireneitega kovblyuki* sp. n.	KY024474	642bp	Tajikstan: Khatlon Area Dangara Distr Sanglogh

Holotypes and some paratypes will be deposited in the Zoological Museum of the Moscow State University
(ZMMU). Most paratypes are deposited in the Institute of Zoology, Chinese Academy of Sciences
(IZCAS) in Beijing, China.

## Taxonomy

### Genus Pireneitega

Taxon classificationAnimaliaAraneaeAgelenidae

Kishida, 1955

Pireneitega Kishida, 1955: 11. Type species Amaurobius roscidus L. Koch, 1868 (= Pireneitega segestriformis (Dufour, 1820)) from Germany.
Paracoelotes Brignoli, 1982: 347. Type species Coelotes armeniacus Brignoli, 1978 from Turkey.

#### Note.

*Pireneitega* was long considered a *nomen nudum* (Yaginuma, in Brignoli 1983: p. 468). Kishida (1955), in a general survey of Agelenidae, considered *Pireneitega* to have been described by himself in 1928, although he had no publications that year. The genus "*Pireneitega* Kishida, 1928 [Genotype: *roscida* (Koch, 1868)]" was considered among the tribe Tegenariini Kishida, 1928 (Kishida 1955: p. 11). Although eye pattern was mentioned in the key to the genera of "Tegenariini", Kishida (1955) did not provide a formal description of the genus. Brignoli (1982) described *Paracoelotes* (type species *Coelotes armeniacus* Brignoli, 1978) from Turkey. Subsequently, Wang and Jäger (2007) revalidated *Pireneitega* with *Paracoelotes* as a junior synonym.

#### Diagnosis.

**Figure 1.**
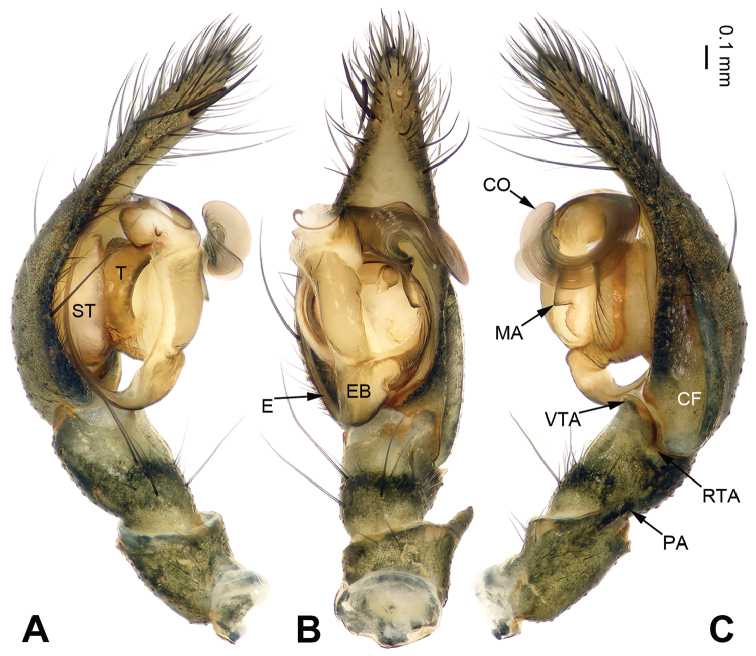
Male palp of *Pireneitega zonsteini* sp. n., holotype. **A** Prolateral **B** Ventral **C** Retrolateral. Scale bar 0.1 mm.

**Figure 2.**
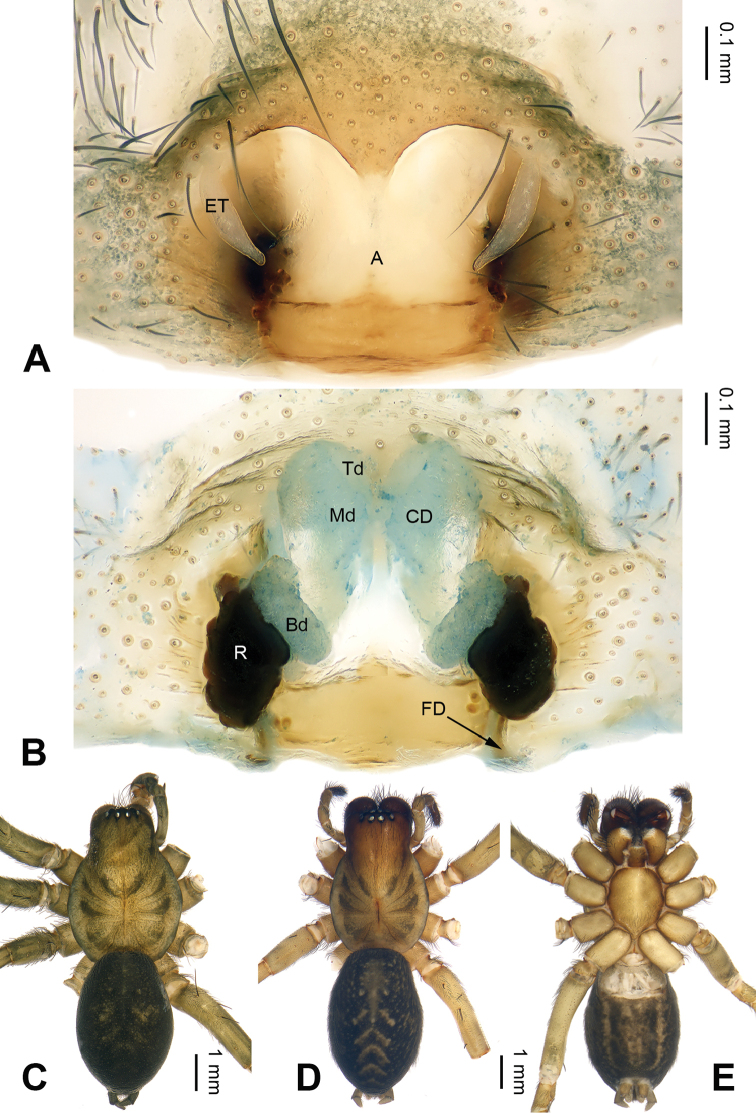
*Pireneitega zonsteini* sp. n., female paratype and male holotype. **A** Epigyne, ventral **B** Vulva, dorsal **C** Male habitus, dorsal **D** Female habitus, dorsal **E** Female habitus, ventral. Scale bars equal for **D, E**.

The chelicerae in most species of *Pireneitega* (including the type species) have 3 promarginal and 3 retromarginal teeth; other coelotines have either 2 or 4 retromarginal teeth (Zhang et al. 2016). The females can be distinguished by the widely separated epigynal teeth, the large atrium with subparallel margins, and the broad copulatory ducts (Fig. 2A–B); other coelotines usually have a small atrium and copulatory ducts. The males can be distinguished by the absence of a dorsal “apophysis” on the conductor, the small RTA, and the distinct median apophysis (Fig. 1A–C); other coelotines usually have a broad dorsal apophysis on the conductor and a reduced or indistinct median apophysis.

#### Composition.

Thirty species of *Pireneitega* are known from Spain to Sakhalin (World Spider Catalog 2016; Mikhailov 2013). One species, *Pireneitega major*, was known from Tajikistan before the current study.

### Pireneitega zonsteini sp. n.

Taxon classificationAnimaliaAraneaeAgelenidae

http://zoobank.org/1AF265B6-AAB0-4974-A8A7-94A906F8FBBF

Figs 1 , 2 , 8

#### Type material.

Holotype ♂ (ZMMU): Tajikstan, environs of Dushanbe, Hissar Mt. Range, 48th km of Varzob Hwy, S exposed slope with *Juglans* litter & under stones, 38°55'31"N, 68°48'18"E, 1530 m, 7.05.2015 (Y.M. Marusik, M. Saidov). Paratypes: 1♂1♀ (IZCAS), same data as holotype.

#### Etymology.

The species is named after Sergei Zonstein (University of Tel-Aviv, Israel) a partner of the junior author in the expedition to Tajikistan; noun (name) in genitive case.

#### Diagnosis.

The male can be distinguished from all other *Pireneitega* species except *Pireneitega involuta* (Wang et al., 1990) by having a broad conductor and thick patellar apophysis. From *Pireneitega involuta* it is distinguished by the blunt tip of the patellar apophysis (*vs* a tapering tip in *Pireneitega involuta*) (Fig. 1; Wang et al. 1990: figs 13–15). The female can be distinguished from all other *Pireneitega* species except *Pireneitega fedotovi* by having a nearly trapezoidal atrium, long copulatory ducts, and short receptacles. From *Pireneitega fedotovi* it can be distinguished by its short epigynal teeth, about 0.5 times as long as length of the atrium (*vs* long epigynal teeth in *Pireneitega fedotovi*, about as long as the length of the atrium) (Fig. 2; Charitonov 1946: fig. 4).

#### Description.

Male (holotype): Total length 8.90. Carapace 4.40 long, 3.50 wide. Abdomen 4.50 long, 2.80 wide. Eye sizes and interdistances: AME 0.15, ALE 0.20, PME 0.15, PLE 0.20; AME-AME 0.07, AME-ALE 0.06, PME-PME 0.15, PME-PLE 0.18. Leg measurements: I: 12.95 (3.50, 4.30, 3.15, 2.00); II: 12.25 (3.25, 4.00, 3.00, 2.00); III: 10.40 (3.15, 3.00, 3.25, 1.00); IV: 16.00 (4.50, 5.00, 4.25, 2.25). Carapace greenish, the radial grooves indistinct, with black lateral margins. Abdomen blackish, with yellow herringbone pattern.

Spination in male

**Table:** Pireneitega zonsteini sp. n. Spination in male

	Fe	Pt	Ti	Mt	Ta
I	3d 2p 1r	–	3-3v	3-3v	–
II	3d 1p 1r	–	2p 3-3v	2p 3-3v	–
III	3d 2p 2r	1p 1r	1d 2p 2r 3-3v	2p 2r 3-3v	–
IV	3d 2p 1r	1p 1r	2p 2r 3-3v	2p 2r 3-3v	–

Palp as in Fig. 1: patellar apophysis long, more than half length of tibia; tibia short, about 1/4 length of tarsus; VTA subequal to the tibial length, without pointed tip, extending beyond the tibia; RTA short, about 1/6 length of VTA; cymbial furrow long, more than half length of cymbium; conductor broad and with two spiraling loops; median apophysis broad and nearly triangular; embolus with broad base originating proximally on base of tegulum.

Female (paratype): Total length 10.0. Carapace 4.75 long, 3.65 wide. Abdomen 5.25 long, 3.45 wide. Eye sizes and interdistances: AME 0.20, ALE 0.25, PME 0.21, PLE 0.26; AME-AME 0.08, AME-ALE 0.05, PME-PME 0.17, PME-PLE 0.20. Leg measurements: I: 12.50 (3.75, 4.25, 2.75, 1.75); II: 11.75 (3.50, 4.00, 2.75, 1.50); III: 10.60 (3.00, 3.50, 2.60, 1.50); IV: 15.00 (4.25, 4.75, 4.00, 2.00). Carapace yellow. Abdomen black, with yellow spots and herringbone pattern.

Epigyne as in Fig. 2A–B: epigynal teeth narrow and relatively short (shorter than width of atrium); septum short with weakly sclerotized tip, about 0.3 times as long as wide; atrium with well delimited posterior margin, about 1.3 times longer than wide, about 4 times longer than septum, subequal to width of septum; copulatory opening hidden by anterior margin of atrium; receptacles long, about 2 times longer than wide, separated by 2.5 times their diameters; copulatory ducts with 3 parts, the basal part running from receptacle posteriorly (*Bd*), median part running anteriorly (*Md*), and terminal part (*Td*) running posteriorly and leading to copulatory opening; median part as wide as terminal and 2 times longer than basal part; median part 1.5 times longer than receptacle; median parts touching each other; hoods indistinct.

Spination in female

**Table:** Pireneitega zonsteini sp. n. Spination in female

	Fe	Pt	Ti	Mt	Ta
I	3d 2p 1r	–	3-3v	3-3v	–
II	3d 1p 1r	–	1p 3-3v	1p 3-3v	–
III	3d 1p 2r	1p 1r	2p 2r 3-3v	2p 2r 3-3v	–
IV	3d 1p 1r	1p 1r	2p 2r 3-3v	1p 2r 3-3v	–

#### Distribution.

Known only from the type locality (Fig. 8).

### Pireneitega muratovi sp. n.

Taxon classificationAnimaliaAraneaeAgelenidae

http://zoobank.org/A01FC654-273B-4E50-A278-052B957FBA4B

Figs 3 , 8

#### Type material.

Holotype ♀ (ZMMU): Tajikstan: env of Dushanbe, Hissar Mt. Ridge, 20^th^ km of Varzob Hwy, Gusgarf [Gushharf] Vill., N exposed slope with *Acer* litter & cliffs, 38°44'22"N, 68°47'33"E, 1750 m, 8.05.2015, Y. M. Marusik. Paratype: 1♀ (IZCAS), same data as holotype.

#### Etymology.

The species is named after Tajikistan zoologist Rustam Muratov (Dushanbe, Tajikistan) who was very helpful in organizing the expedition to Tajikistan; noun (name) in genitive case.

#### Diagnosis.

**Figure 3.**
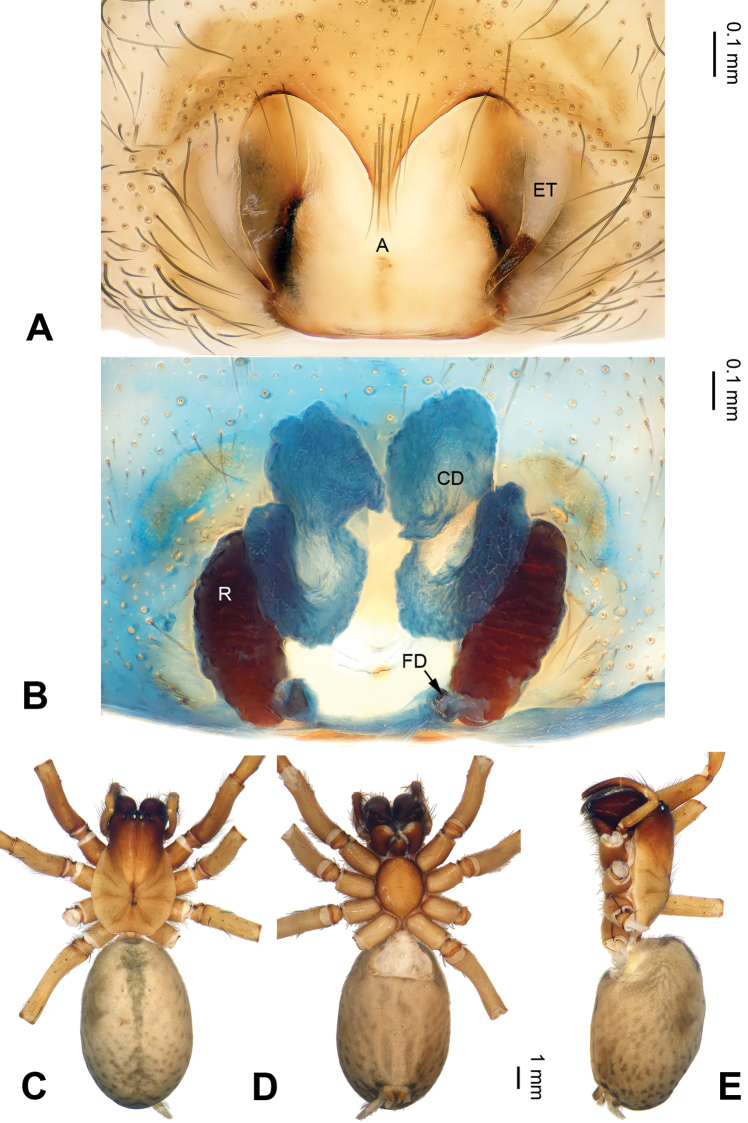
*Pireneitega muratovi* sp. n., female holotype. **A** Epigyne, ventral **B** Vulva, dorsal **C** Habitus, dorsal **D** Habitus, dorsal **E** Habitus, ventral view. Scale bars equal for **C, D, E**.

The female can be distinguished from all other *Pireneitega* species except *Pireneitega fedotovi*, *Pireneitega luniformis* (Zhu & Wang, 1994), and *Pireneitega major* by having narrow epigynal teeth and an elongate oval atrium. It can be distinguished from *Pireneitega fedotovi* by the pointed tip of septum (*vs* blunt tip in *Pireneitega fedotovi*), from *Pireneitega luniformis* by the elongate oval receptacles (*vs* spiralled in *Pireneitega luniformis*), and from *Pireneitega major* by its short epigynal teeth, *ca.* 0.8 times as long as length of the atrium (*vs* long epigynal teeth in *Pireneitega major*, about as long as the length of the atrium) (Figs 3, 7; Charitonov 1946: fig. 4; Zhu and Wang 1994: figs 5–6).

#### Description.

Male: unknown.

Female (holotype): Total length 9.94. Carapace 4.49 long, 3.05 wide. Abdomen 5.45 long, 2.90 wide. Eye sizes and interdistances: AME 0.18, ALE 0.23, PME 0.24, PLE 0.30; AME-AME 0.10, AME-ALE 0.05, PME-PME 0.15, PME-PLE 0.10. Leg measurements: I: 11.25 (3.25, 4.00, 2.50, 1.50); II: 10.30 (3.00, 3.50, 2.50, 1.30); III: 9.70 (2.75, 3.00, 2.65, 1.30); IV: 13.75 (3.75, 4.25, 4.00, 1.75). Carapace yellow, the radial grooves indistinct. Abdomen whitish-yellow, with green herringbone pattern.

Epigyne as in Fig. 3A–B: epigynal teeth narrow, their length equal to width of the narrowest part of the atrium; septum with well delimited tip, *ca.* 0.5 times as long as wide; copulatory opening distinct; atrium with well delimited posterior margin, about 1.4 times longer than wide, *ca.* 2 times longer than and 0.7 times as wide as septum; receptacles long, about 2.5 times as long as wide, bases of receptacles separated by 2 diameters; copulatory ducts with 3 parts, median part as long as receptacles, and anterior part slightly wider than receptacles; hoods indistinct.

Spination

**Table:** Pireneitega muratovi sp. n. Spination

	Fe	Pt	Ti	Mt	Ta
I	3d 2p 1r	–	3-3v	1p 3-3v	–
II	3d 3p 2r	–	2p 3-3v	3-3v	–
III	3d 3p 2r	1p 1r	2p 2r 3-3v	5p 4r 3-3v	1p 1r
IV	3d 1p 1r	1d 1p 1r	1d 2p 2r 3-3v	5p 5r 3-3v	2p 1r

#### Distribution.

Known only from the type locality (Fig. 8).

### Pireneitega tyurai sp. n.

Taxon classificationAnimaliaAraneaeAgelenidae

http://zoobank.org/B14F37A9-6A33-446F-80AF-2C65472362D3

Figs 4 , 8

#### Type material.

Holotype ♀ (ZMMU): Tajikstan: Khatlon Area, Khovaling Distr., Obimazar River, Sultan-Mazar, clay cliffs, 38°28'19"N, 70°04'01"E, 1854 m, 27.04.2015 (Y.M. Marusik). Paratypes: 4♀ (IZCAS), same data as holotype.

#### Etymology.

The species is named after Sergei V. Tyura (Magadan, Russia) a friend of the junior author; noun (name) in genitive case.

#### Diagnosis.

**Figure 4.**
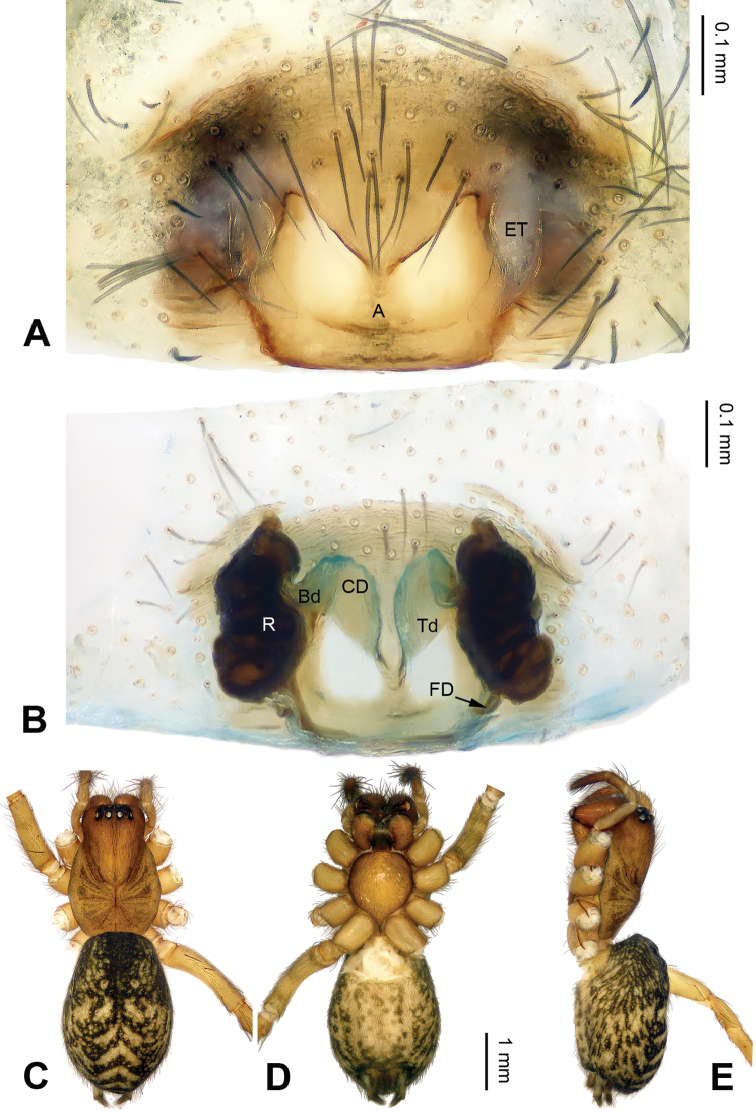
*Pireneitega tyurai* sp. n., female holotype. **A** Epigyne, ventral **B** Vulva, dorsal **C** Habitus, dorsal **D** Habitus, dorsal **E** Habitus, ventral. Scale bars equal for **C, D, E**.

The female can be distinguished from all other *Pireneitega* species except *Pireneitega tianchiensis* (Wang et al., 1990) by having short receptacles and copulatory ducts. It can be distinguished from *Pireneitega tianchiensis* by the broad and long epigynal teeth, about 0.85 times as long as atrium (*vs* short and narrow epigynal teeth in *Pireneitega tianchiensis*, about 0.5 times as long as atrium) (Fig. 4A–B; Wang et al. 1990: figs 84–85).

#### Description.

Male: unknown.

Female (holotype): Total length 5.15. Carapace 2.15 long, 1.75 wide. Abdomen 3.00 long, 2.00 wide. Eye sizes and interdistances: AME 0.10, ALE 0.13, PME 0.15, PLE 0.15; AME-AME 0.05, AME-ALE 0.10, PME-PME 0.02, PME-PLE 0.04. Leg measurements: I: 6.20 (1.90, 2.25, 1.25, 0.80); II: 5.10 (1.60, 1.75, 1.00, 0.75); III: 4.80 (1.50, 1.60, 1.00, 0.70); IV: 7.05 (2.05, 2.50, 1.50, 1.00). Carapace yellow, with black lateral margins. Abdomen blackish, with yellow herringbone pattern.

Epigyne as in Fig. 4A–B: epigynal teeth long (nearly as long as atrium); septum with weakly sclerotized tip, about 0.5 times as long as wide; atrium with weakly sclerotized posterior margin, about 0.7 times as long as wide, about 1.8 times longer than and 0.7 times as wide as septum; copulatory opening hidden; receptacles large, *ca.* 2 times longer than wide; copulatory ducts with two parts, terminal parts (Tp) not touching each other, about 0.5 length of receptacles, basal parts (Bp) shorter than width of receptacle; hoods indistinct.

Spination

**Table:** Pireneitega tyurai sp. n. Spination

	Fe	Pt	Ti	Mt	Ta
I	3d 2p	–	3-3v	3-3v	–
II	3d 1p 1r	1p	2p 3-3v	1p 3-3v	–
III	3d 1p 1r	1p 1r	2p 2r 3-3v	5p 4r 3-3v	2p 1r
IV	2d 1p 1r	1p 1r	2p 2r 3-3v	5p 4r 3-3v	2p 1r

#### Distribution.

Known only from the type locality (Fig. 8).

### Pireneitega ramitensis sp. n.

Taxon classificationAnimaliaAraneaeAgelenidae

http://zoobank.org/C74C6BAE-DE7C-4A95-A4A2-5E5BFC45C341

Figs 5 , 8

#### Type material.

Holotype ♀ (ZMMU): Tajikstan: Khatlon Area, Hissar Mt. Range, Ramit Reserve, 38°44'36"N, 69°18'30"E, 1324 m, 1.05.2015 (Y.M. Marusik). Paratypes: 4♀ (IZCAS), 2♀ (ZMMU), same data as holotype.

#### Etymology.

The specific name is an adjective and refers to the type locality; adjective.

#### Diagnosis.

**Figure 5.**
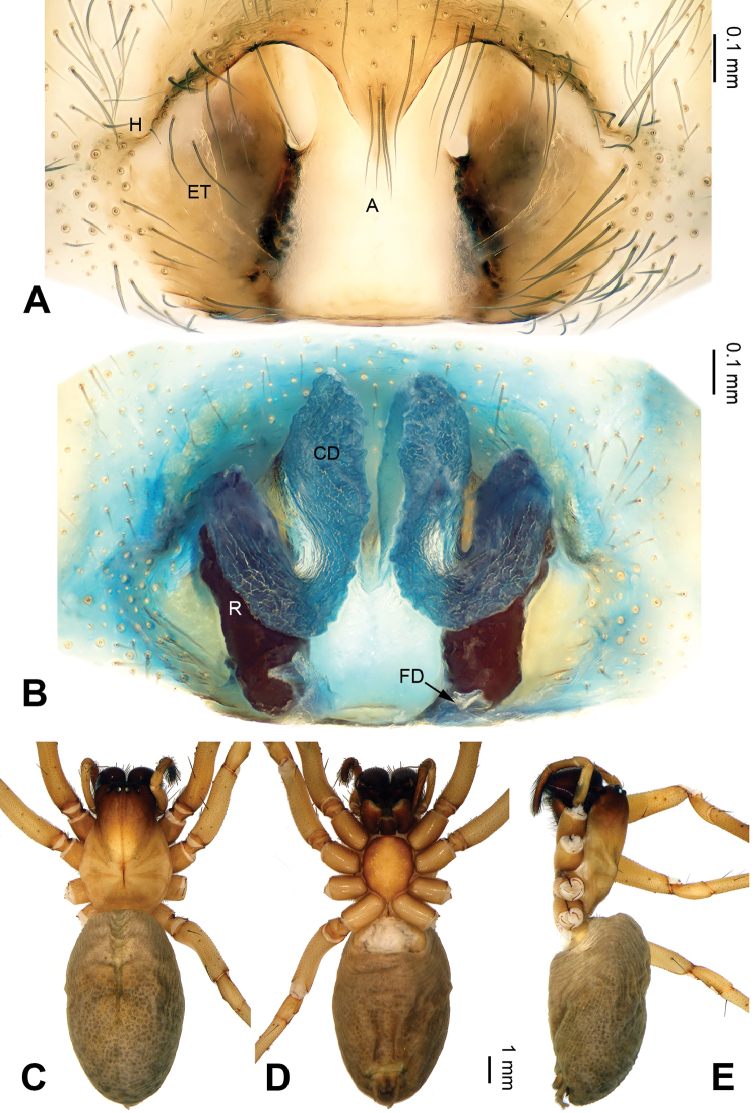
*Pireneitega ramitensis* sp. n., female holotype. **A** Epigyne, ventral **B** Vulva, dorsal **C** Habitus, dorsal **D** Habitus, ventral **E** Habitus, ventral. Scale bars equal for **C, D, E**.

The female can be distinguished from all other *Pireneitega* species except *Pireneitega muratovi* sp. n., *Pireneitega fedotovi*, *Pireneitega luniformis* and *Pireneitega major*, by having an elongate oval atrium, narrow epigynal teeth, and long copulatory ducts. It can be distinguished from *Pireneitega muratovi* sp. n. and *Pireneitega luniformis* by the narrow tip of the copulatory ducts (*vs* round tip in *Pireneitega muratovi* sp. n. and *Pireneitega luniformis*) and from *Pireneitega fedotovi* and *Pireneitega major* by the bent epigynal teeth (*vs* straight epigynal teeth in *Pireneitega fedotovi* and *Pireneitega major*) (Figs 3, 5, 7; Charitonov 1946: fig. 4; Zhu and Wang 1994: figs 5–6).

#### Description.

Male: unknown.

Female (holotype): Total length 12.00. Carapace 4.50 long, 3.55 wide. Abdomen 7.50 long, 4.75 wide. Eye sizes and interdistances: AME 0.20, ALE 0.23, PME 0.25, PLE 0.20; AME-AME 0.10, AME-ALE 0.20, PME-PME 0.10, PME-PLE 0.23. Leg measurements: I: 14.05 (4.00, 4.75, 3.45, 1.85); II: 13.40 (3.90, 4.50, 3.25, 1.75); III: 13.00 (3.75, 4.25, 3.25, 1.75); IV: 16.55 (4.75, 5.40, 4.40, 2.00). Carapace yellowish, with brown lateral margins. Abdomen pale-yellow, with brown spots.

Epigyne as in Fig. 5A–B: epigynal teeth pale, hyaline, long and thin, about 0.9 times as long as receptacles; septum with weakly sclerotized tip, *ca.* 0.5 times as long as wide, nearly triangular; copulatory ducts distinct; atrium about 1.4 times longer than wide, with well delimited posterior margin, *ca.* 2.8 times longer than and about as wide as septum; receptacles large, about. 3 times longer than wide; receptacle bases separated by about 2 diameters; copulatory ducts with 3 parts, basal part about 2/3 of receptacle length, median part as long as receptacle, terminal part somewhat shorter than median part; hoods distinct.

Spination

**Table:** Pireneitega ramitensis sp. n. Spination

	Fe	Pt	Ti	Mt	Ta
I	3d 2p 1r	–	1p 3-3v	1p 3-3v	–
II	3d 2p 2r	–	2p 3-3v	2p 3-3v	–
III	3d 3p 2r	1p 1r	2p 2r 3-3v	5p 4r 3-3v	2p 2r
IV	3d 2p 1r	1p 1r	2p 2r 3-3v	5p 4r 3-3v	2p 2r

#### Distribution.

Known only from the type locality (Fig. 8).

### Pireneitega kovblyuki sp. n.

Taxon classificationAnimaliaAraneaeAgelenidae

http://zoobank.org/25787234-B768-4EB3-B6B2-781E025AB5D4

Figs 6 , 8

#### Type material.

Holotype ♂ (ZMMU): Tajikstan, Khatlon Area, Dangara Distr., Sanglogh (=Sanglok) Mt. Range above Shar-Shar Pass, 38°17'56"N, 69°13'36"E, 1700–2060 m, 29.04.2015, (Y.M. Marusik). Paratypes: 3♂ (IZCAS), 2♂ (ZMMU), same data as holotype.

#### Etymology.

The specific name is a patronym in honour of the well known arachnologist and friend of the junior author Mykola M. Kovblyuk (Simferopol, Ukraine); noun (name) in genitive case.

#### Diagnosis.

**Figure 6.**
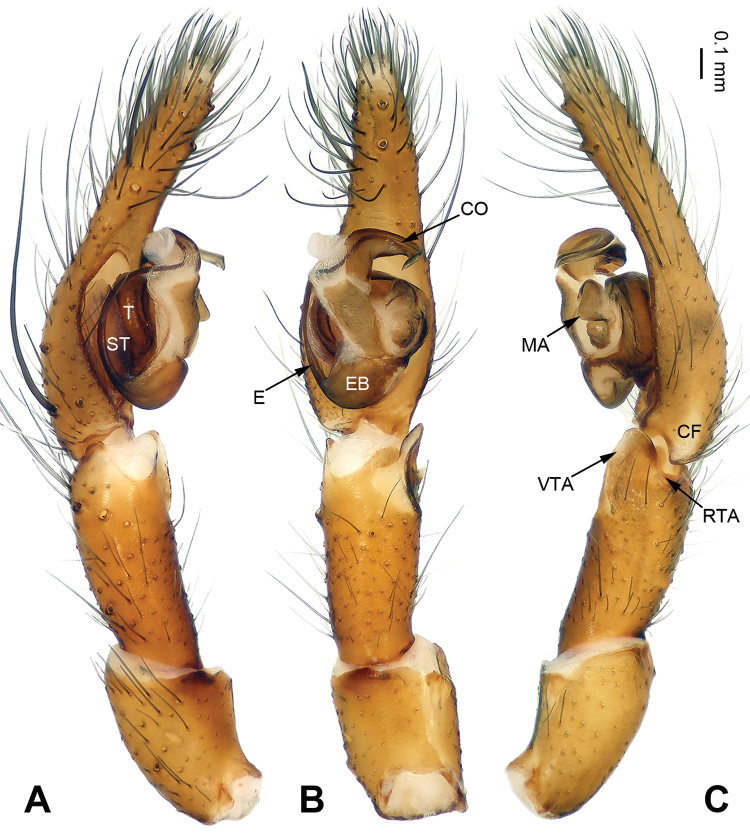
Male palp of *Pireneitega kovblyuki* sp. n., holotype. **A** Prolateral **B** Ventral **C** Retrolateral. Scale bar 0.1 mm.

The male can be distinguished from all other *Pireneitega* species except *Pireneitega tianchiensis* by having a hook-shaped conductor, and narrow cymbium. It can be distinguished from *Pireneitega tianchiensis* by the short cymbial furrow, *ca.* 1/10 length of cymbium (*vs* long cymbial furrow in *Pireneitega tianchiensis*, about 0.5 length of cymbium) (Fig. 6; Wang et al. 1990: figs 81–83).

#### Description.

Male (holotype): Total length 7.90. Carapace 4.00 long, 3.00 wide. Abdomen 3.90 long, 2.65 wide. Eye sizes and interdistances: AME 0.15, ALE 0.20, PME 0.18, PLE 0.19; AME-AME 0.08, AME-ALE 0.07, PME-PME 0.13, PME-PLE 0.15. Leg measurements: I: 10.90 (3.25, 4.05, 2.00, 1.60); II: 9.85 (3.00, 3.50, 2.00, 1.35); III: 8.60 (2.75, 2.50, 2.10, 1.25); IV: 12.55 (3.70, 3.75, 3.50, 1.60). Carapace yellow, the radial grooves indistinct. Abdomen pale, with yellow herringbone pattern.

Palp as in Fig. 6A–C: patellar apophysis absent; tibia long, *ca.* 0.5 length of cymbium; VTA short and wide, about 1/3 length of tibia, without pointed tip; RTA short, about 1/5 length of VTA, poorly visible; cymbium long, its tip as long as or longer than genital bulb; conductor short, with hook-shaped, partially looped tip, tip located distally from tegulum; median apophysis broad and nearly triangular; embolus with broad, nearly tongue-shaped base, beginning at 6:30 o’clock position.

Spination

**Table:** Pireneitega kovblyuki sp. n. Spination

	Fe	Pt	Ti	Mt	Ta
I	3d 2p 1r	–	3-3v	3-3v	–
II	3d 3p 1r	1p	2p 3-3v	3p 3-3v	–
III	3d 2p 2r	1d1p 1r	1d 2p 2r 3-3v	5p 5r 3-3v	1p 1r
IV	3d 1p 1r	1p 1r	2p 2r 3-3v	5p 5r 3-3v	2p 1r

Female: Unknown.

#### Distribution.

Known only from the type locality (Fig. 8).

### Pireneitega major

Taxon classificationAnimaliaAraneaeAgelenidae

(Kroneberg, 1875)

Figs 7 , 8

Coelotes major Kroneberg, 1875: 15, pl. 1, fig. 6 (♀); Charitonov, 1946: 20, fig. 5 (♀).
Paracoelotes major : Ovtchinnikov, 1988: 142 (transferred from Coelotes).
Coelotes major : Schenkel 1936: 284, fig. 97 (♀); Hu & Wu 1989: 180, fig. 150.1‒2 (♀). Misidentification
Paracoelotes major : Song et al. 1999: 389, fig. 229Q‒R (♀). Misidentification

#### Material examined.

Lectotype ♀ (ZMMU) with label «Ta 3845 1♀ ZMMU [Зоомузей МГУ]» «Lectotypus» 2/VI; Аyчи дагана [Auchi dagana] *Coelotes major* Kroneberg, 1875», ca 39°35’N, 69°05’E. Paralectotype: 1♀ (ZMMU) with 2 labels «Ta1059, 1, *Coelotes major*» «Туркестанская Учёная Экспедиция Имераторскаго Общества Любителей Естествознанiя. Федченко [Turkestan Scientific Expedition of the Emperor’s Society of Devotees of Natural Sciences. Fedchenko]» and «*Coelotes major* n. sp. Ta, No.1059, Кокандское ханство, Федченко [Kokand Khanate, Fedchenko]».

#### Comments.

The figures of *Pireneitega major* presented by Schenkel (1936), Hu and Wu (1989), and Song et al. (1999; copied from Hu and Wu 1989) are of a species other than *Pireneitega major*, the identity of which is currently unknown. All records of this unknown species are from Xinjiang, China.

#### Diagnosis.

**Figure 7.**
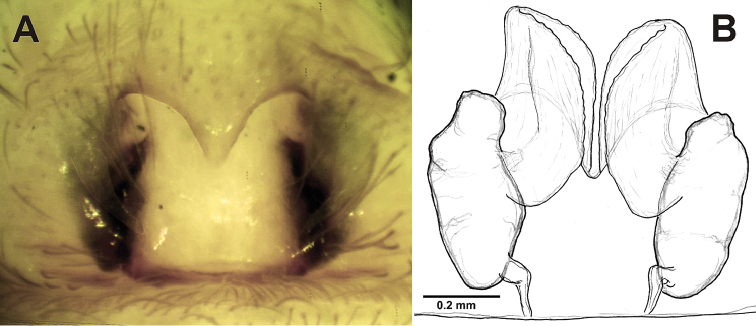
Epigyne of *Pireneitega major*, lectotype. **A** Ventral **B** Dorsal.

This species is easily distinguished from other species of *Pireneitega* found in Tajikistan by its larger size (carapace length >6 mm *vs* <4.75) and having 5 spines on tarsus IV (*vs* other species with 0‒4). The epigyne of *Pireneitega major* is most similar to that of *Pireneitega muratovi* sp. n. and *Pireneitega ramitensis* sp. n. It can be distinguished from *Pireneitega muratovi* sp. n. by its shorter receptacles with length/width ratio of 2.3 (*vs* 2.6 in *Pireneitega muratovi*), shape of copulatory ducts, and shorter teeth (cf. Figs 3A–B and 7A–B). *Pireneitega major* can be separated from *Pireneitega ramitensis* sp. n by its wider epigynal atrium and shorter, wider receptacles as well as by its shorter and wider copulatory ducts (cf. Figs 5A–B and 7A–B).

#### Description.

Male: unknown.

Female: Lectotype. Total length 16.7. Carapace 7.0 long, 5.0 wide, fovea 1.25 long. Leg measurements: I:19.75 (5.5, 2.5, 4.6, 4.65, 2.5); II: 18.6 (5.1, 2.5, 4.0, 4.5, 2.5); III: 17.2 (4.75, 2.2, 3.55, 4.6, 2.1); IV: 21.85 (5.75, 2.3, 5.0, 6.25, 2.55).

Spination

**Table:** Pireneitega major (Kroneberg, 1875) Spination

	Fe	Pt	Ti	Mt	Ta
I	3d 2p 2r	–	3-3v	3-3v 1vm	–
II	3d 3p 2r	–	2p 3-3v	1p 3-3v	–
III	3d 3p 2r	1p 1(0)r	2p 2r 3-3v	5p 4r 3-3v	2p 1-1v
IV	3d 2p 1r	1p 1r	2p 2r 3-3v	5p 4r 3-3v	2p 3r

Paralectotype ♀. Total length: 11.0. Carapace 6.0 long, 4.0 wide. Epigyne 0.51 wide.

Epigyne as in Fig. 7: epigynal teeth pale, hyaline, long and thin; septum with weakly sclerotized tip, about 0.4 times as long as wide, subtriangular; atrium as long as wide; receptacles large, about 2.5 times longer than wide; receptacle bases separated by *ca.* 2 diameters; copulatory ducts with 2 parts, basal part as long as receptacle, terminal part somewhat shorter receptacle.

#### Comments.

**Figure 8.**
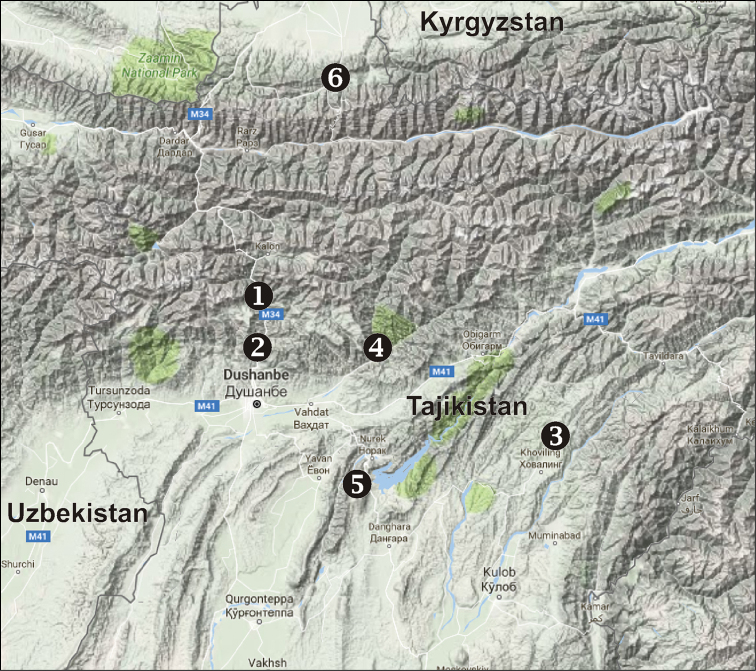
Localities of *Pireneitega* species from Tajikistan. **1** *Pireneitega zonsteini* sp. n. **2** *Pireneitega muratovi* sp. n. **3** *Pireneitega tyurai* sp. n. **4** *Pireneitega ramitensis* sp. n. **5** *Pireneitega kovblyuki* sp. n. **6** *Pireneitega major*.

Known from the type series females only. Exact locality is known for the lectotype only: Auchi lies on the northern macroslope of the Turkestan Mt Range (Fig. 8).

## Supplementary Material

XML Treatment for Pireneitega

XML Treatment for Pireneitega zonsteini

XML Treatment for Pireneitega muratovi

XML Treatment for Pireneitega tyurai

XML Treatment for Pireneitega ramitensis

XML Treatment for Pireneitega kovblyuki

XML Treatment for Pireneitega major
